# Chondrocyte-specific genomic editing enabled by hybrid exosomes for osteoarthritis treatment

**DOI:** 10.7150/thno.69368

**Published:** 2022-06-21

**Authors:** Yujie Liang, Xiao Xu, Limei Xu, Zoya Iqbal, Kan Ouyang, Huawei Zhang, Chunyi Wen, Li Duan, Jiang Xia

**Affiliations:** 1 Department of Orthopedics, the First Affiliated Hospital of Shenzhen University, Shenzhen Second People's Hospital, Shenzhen 518035, China.; 2 Department of Chemistry, the Chinese University of Hong Kong, Shatin, Hong Kong SAR, China.; 3 Department of Biomedical Engineering, South University of Science and Technology of China, Shenzhen, 518055, China.; 4 Department of Biomedical Engineering, The Hong Kong Polytechnic University, Hong Kong SAR, China.; 5 Research Institute of Smart Ageing, The Hong Kong Polytechnic University, Hong Kong SAR, China.

**Keywords:** Therapeutic genome editing, hybrid exosome, CRISPR/Cas9, osteoarthritis, cartilage, MMP-13

## Abstract

**Rationale:** A cell-specific delivery vehicle is required to achieve gene editing of the disease-associated cells, so the hereditable genome editing reactions are confined within these cells without affecting healthy cells. A hybrid exosome-based nano-sized delivery vehicle derived by fusion of engineered exosomes and liposomes will be able to encapsulate and deliver CRISPR/Cas9 plasmids selectively to chondrocytes embedded in articular cartilage and attenuate the condition of cartilage damage.

**Methods:** Chondrocyte-targeting exosomes (CAP-Exo) were constructed by genetically fusing a chondrocyte affinity peptide (CAP) at the N-terminus of the exosomal surface protein Lamp2b. Membrane fusion of the CAP-Exo with liposomes formed hybrid CAP-exosomes (hybrid CAP-Exo) which were used to encapsulate CRISPR/Cas9 plasmids. By intra-articular (IA) administration, hybrid CAP-Exo/Cas9 sgMMP-13 entered the chondrocytes of rats with cartilage damages that mimicked the condition of osteoarthritis.

**Results:** The hybrid CAP-Exo entered the deep region of the cartilage matrix in arthritic rats on IA administration, delivered the plasmid Cas9 sgMMP-13 to chondrocytes, knocked down the matrix metalloproteinase 13 (*MMP-13*), efficiently ablated the expression of *MMP-13* in chondrocytes, and attenuated the hydrolytic degradation of the extracellular matrix proteins in the cartilage.

**Conclusion:** Chondrocyte-specific knockdown of *MMP-13* mitigates or prevents cartilage degradation in arthritic rats, showing that hybrid CAP-Exo/Cas9 sgMMP-13 may alleviate osteoarthritis.

## Introduction

Since its invention in 2012, the Clustered Regularly Interspaced Short Palindromic Repeats (CRISPR) technology has revolutionized gene editing, impacted biotechnology, and brought hope to the treatment of genetic diseases. Genome editing has shown therapeutic potential for treating cancers, blood disorders, blindness, AIDS, cystic fibrosis, muscular dystrophy, Huntington's disease, and, more recently, even Covid-19 1-7. Notwithstanding the translational potential, clinical applications of the CRISPR technology still face potential risks such as off-target effects, immune reactions and increased risks of cancers, etc.

Delivery of the CRISPR/Cas9 system constructed *ex vivo* precisely into the disease-associated cells and organisms may minimize these side effects. Exosomes, cell-derived vesicles with diameters of 30-150 nm that naturally mediate the communication and transportation of cellular contents between cells, have been employed as the carrier for the CRISPR/Cas9 system 8-12. Exosomes are not designed to deliver plasmids; they must be engineered to function as efficient delivery vehicles for large molecules. Increasing the loading efficiency of the CRISPR/Cas9 system into exosomes has been one of the focal points. This can be achieved by engineering protein-protein or protein-RNA interacting pairs on exosomes and cargos, respectively 13-15. Or, the hybridization of exosomes and liposomes forms hybrid nano-vesicles that can load significantly larger molecules with a higher amount than exosomes while maintaining the surface property of exosomes 16, 17. On the other hand, exosomes can be harnessed to achieve targeted delivery of the cargo to only disease-associated cells through the cancer tropism effects or specific cell-surface receptors. For example, Kim and coworkers reported that cancer-derived exosomes could transport the CRISPR/Cas9 system specifically to tumor cells *via* natural exosome-cell interactions 12. Previously, we and others have engineered the exosome-surface protein to display cell-targeting peptides and achieve selective delivery of cargos to chondrocytes or mesenchymal stem cells 18-20.

Gene therapy has now been considered a viable method for treating osteoarthritis (OA). OA is a chronic disease associated with the degeneration of joints such as knees, hips, shoulders, and the spine 21. Increasingly prevalent but facing limited therapeutic options, OA has been defined by World Health Organization as a priority disease in need of research for potential therapies 22. Current therapeutics such as anti-inflammatory and analgesic drugs and steroids provide only short-term alleviation of the symptoms but fail to stop the degenerative process 23-25 or have significant side effects during prolonged use 26, 27. Surgery and joint replacement are expensive and highly invasive 22, 43. As several genes in chondrocytes, the cells residing in the cartilage, have been identified to be tightly associated with the pathology of OA, genome editing is believed to be a potential treatment that provides a long-term benefit to OA patients 28, 29.

**Figure 1:**
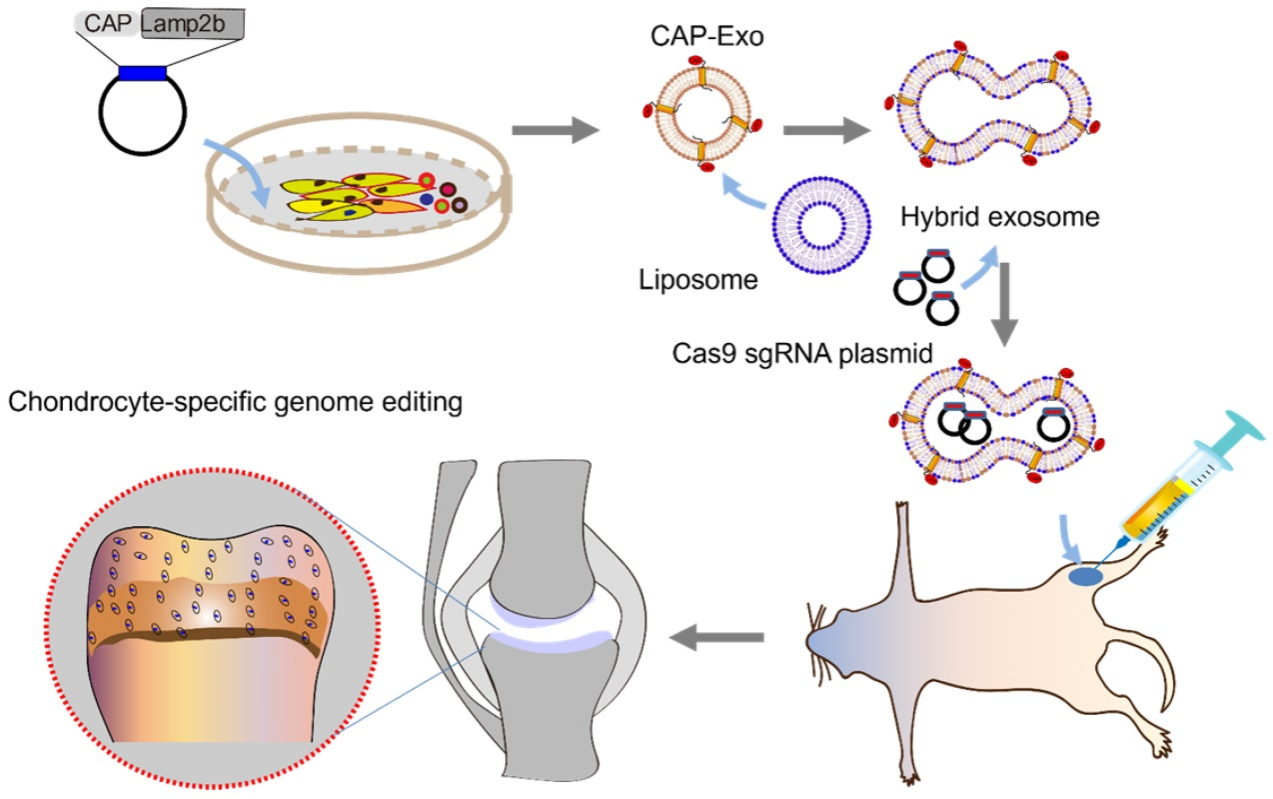
Schematic illustration of chondrocyte-specific genome editing by hybrid exosomes.

Matrix metalloproteinase 13 (*MMP-13*) is one of the therapeutic targets. During the early stage of OA development, overexpression of *MMP-13* causes extracellular matrix degradation, leading to cartilage degeneration 30. Knocking down *MMP-13* or suppressing the enzymatic activity using small molecule inhibitors have shown beneficial effects in preventing or reversing cartilage degeneration in OA 31. For example, intra-articular (IA) injection of *MMP-13*-specific siRNA suppressed the expression of *MMP-13* and delayed cartilage degradation *in vivo* 32, 33. Delivering the therapeutic agents to chondrocytes, however, is a challenge. The cartilage matrix often rejects the therapeutics, and the synovial fluid in the joint flushes away drugs administered to the joint. The dense cartilage matrix poses a thick physical barrier and impedes the infiltration of biomolecular medications 34, 35. Encapsulation of the therapeutics in nano-sized carriers can protect the molecules from unwanted degradation, increase the penetration into the cartilage matrix and increase the bioavailability 36, 37. Here we report a chondrocyte-specific IA delivery of a genome editing tool, a CRISPR/Cas9 plasmid, explicitly targeting the *MMP-13* by hybrid exosome derived by fusion of exosomes and liposome to treat OA in a rat model (Figure 1).

## Results

### Chondrocyte-targeting hybrid exosomes

**Figure 2:**
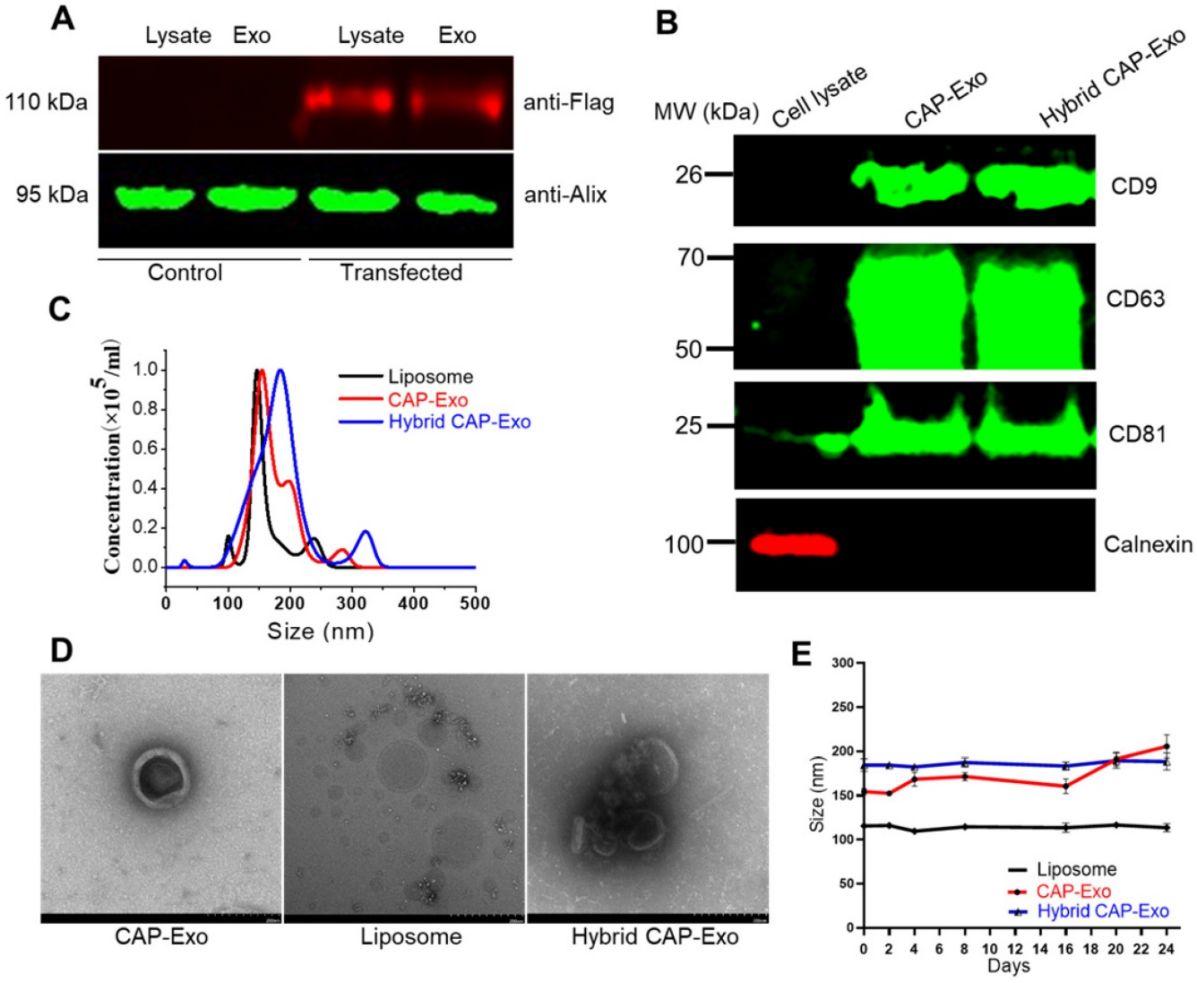
** Characterization of the exosomes. (A)** Western blotting analysis of DC lysate and DC-derived exosome, un-transfected or transfected with a CAP-lamp2b plasmid carrying a FLAG tag. **(B)** Immunoblots of cell lysates, CAP-Exo, and hybrid CAP-Exo show the expression of exosome markers (CD9, CD81, and CD63). **(C)** Hydrodynamic sizes of CAP-Exo, liposome, and hybrid CAP-Exo measured by NTA analysis. **(D)** TEM images of CAP-Exo, liposome, and hybrid CAP-Exo. Scale bar: 200 nm. **(E)** Stability of CAP-Exo, liposome, and hybrid CAP-Exo measured by the size change over 24 d at 4 °C.

We first developed chondrocyte-targeting exosomes, which contain a surface-displayed chondrocyte-affinity peptide (CAP) through genetic engineering of the exosomal membrane protein, lysosomal-associated membrane protein 2b (Lamp2b) in dendritic cells (DC). Because the N-terminus of Lamp-2b extends out of the exosome membrane, fusing a targeting peptide at this end does not compromise the expression and function of Lamp2b 38, 39. A CAP-FLAG-Lamp2b plasmid was constructed and delivered into dendritic cells. A FLAG tag was inserted between the CAP sequence and Lamp2b for a facile tracing of the exosomes. Exosomes purified by ultracentrifugation from the genetically modified dendritic cells carry the CAP-FLAG-Lamp2b protein and are thus called CAP-Exo. To house the CRISPR/Cas9 plasmid, we next constructed hybrid exosomes by fusing CAP-Exo with liposomes formed by Lipofectamine 2000. The resultant hybrid CAP-Exo vesicles contain exosomal marker proteins CD9, CD81, and CD63, similar to exosomes (Figures 2A-B), while having a larger size than exosome or liposome, based on nanoparticle tracking analysis (NTA) and transmission electron microscope (TEM) (Figures 2C-D). The hybrid CAP-Exo also showed higher stability than exosomes as their size remained constant for at least 24 days, whereas exosomes gradually aggregated (Figure 2E). The hybridization of exosomes and liposomes also changed the zeta potential of the particles (Figure S1). The fusion of exosomes and liposomes was also supported by a FRET assay (Figure S2). Therefore, membrane fusion of exosomes with liposomes results in hybrid exosomes with larger sizes, higher stability, and yet the same surface proteins.

### Intracellular uptake of CAP-Exo by chondrocytes

**Figure 3:**
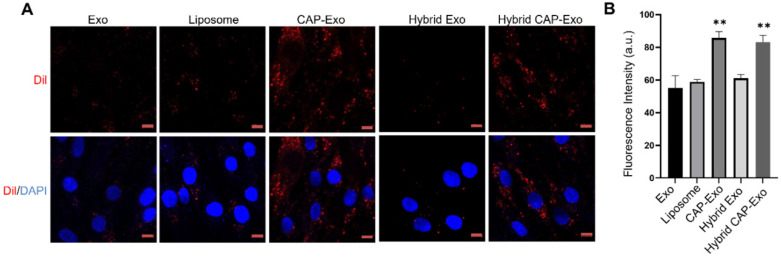
** Intracellular uptake by chondrocytes. (A)** Confocal images of chondrocytes envisage the internalization of DiI-labeled exosomes, liposomes, or hybrid exosomes. Briefly, rat chondrocytes were incubated with DiI-labeled vesicles (red) at 10^8^/mL for 2 h and imaged using a confocal laser scanning microscope. Cell nuclei were labeled by DAPI (blue). Data represent the mean ± SD from three independent experiments. **p < 0.01 compared to the Exo group. Scale bar, 10 µm. **(B)** Quantification of the mean intensity of the fluorescent signals using Image J.

To determine if hybrid CAP-Exo could efficiently enter primary chondrocytes, we fluorescently labeled the exosomes or liposomes with DiI, a long-chain lipophilic carbocyanine dye that labels membrane-containing vesicles. When the DiI-labeled hybrid CAP-Exo were incubated with primary chondrocytes, fluorescent signals were observed inside chondrocytes showing a successful uptake of the vesicles in the cells. Cells treated with vesicles carrying the CAP sequence (CAP-Exo and hybrid CAP-Exo groups) showed significantly higher signals than cells treated with exosomes without surface-functionalized CAP sequence (Exo group) or hybrid exosomes without CAP (hybrid Exo group) (Figures 3A-B). Together with the data from flow cytometry (Figure S3), these results indicate that the CAP sequence plays a critical role in the uptake of exosomes in chondrocytes.

### Knockdown of the MMP-13 gene in chondrocytes

**Figure 4:**
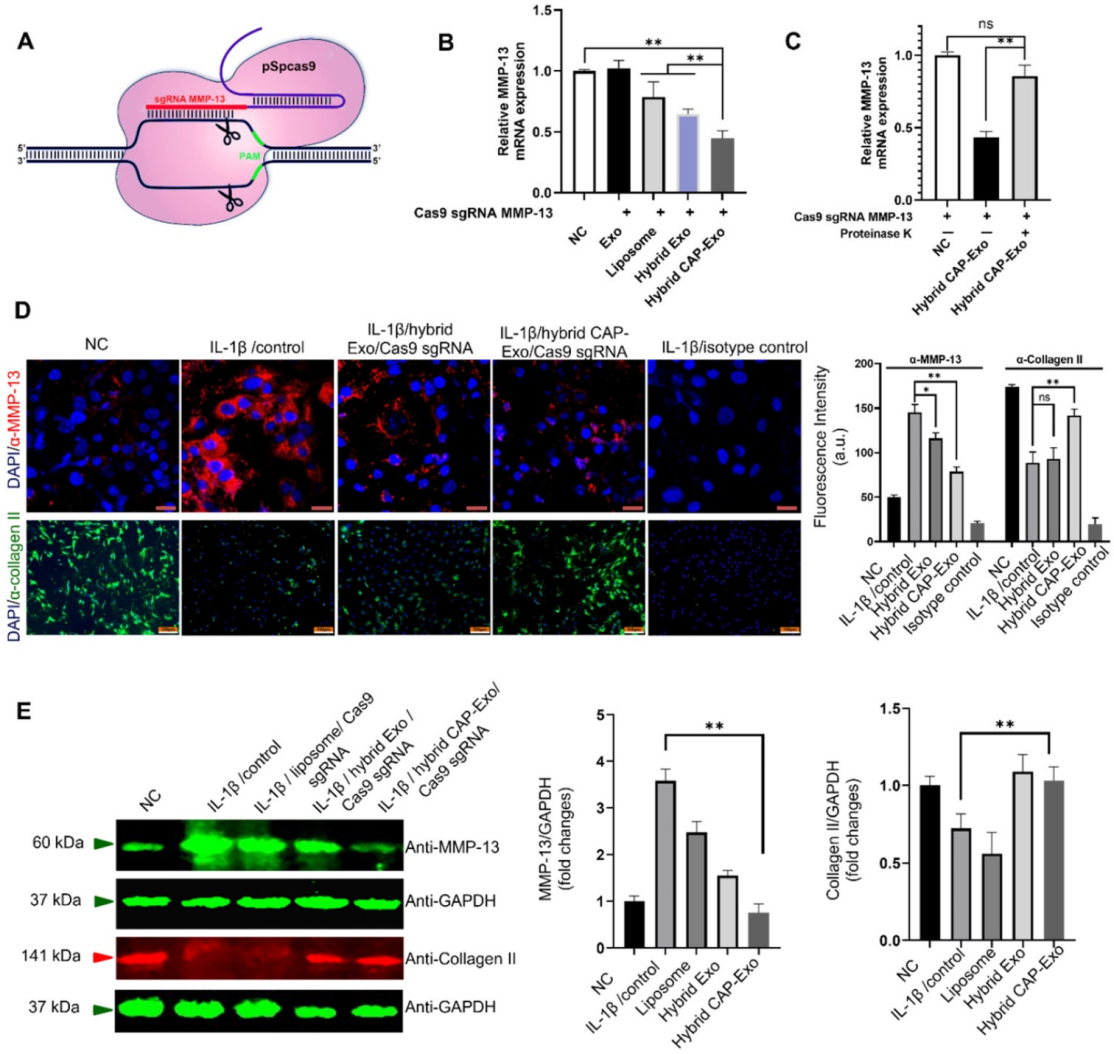
** Exosome-mediated delivery of the Cas9 sgMMP-13 to knock down *MMP-13* in chondrocytes. (A)** Schematic illustration of the design of the Cas9 sgMMP-13 system. **(B)** *MMP-13* mRNA level measured by qRT-PCR in rat chondrocytes incubated with Cas9 sgMMP-13 only (negative control group, NC), exosome/Cas9 sgMMP-13 (Exo), liposome/Cas9 sgMMP-13 (liposome), and hybrid Exo/Cas9 sgMMP-13 (hybrid Exo), hybrid CAP-Exo/Cas9 sgMMP-13 (hybrid CAP-Exo). **(C)** qRT-PCR analysis of *MMP-13* mRNA level in the chondrocytes treated with hybrid CAP-Exo/Cas9 sgMMP-13 with and without proteinase K treatment. **(D)** Upper panel, representative immunofluorescence images of the MMP-13 expression levels in different treatment groups using an anti-MMP-13 antibody, scale bar: 20 µm. Lower panel, hybrid CAP-Exo/Cas9 sgMMP-13 attenuated type II collagen degradation based on immunofluorescence imaging using a collagen II antibody. Isotype controls did not show staining, scale bar, 100 µm. Quantification of the fluorescent signals is shown on the right side. **(E)** Western blotting of the* MMP-13* and collagen II expression levels in different treatment groups after 48 h and quantification of the fluorescent signals. Data were expressed as mean ± SEM of three different experiments. * *p* < 0.05, ** *p*< 0.01.

*MMP-13* is a protease responsible for the degradation of the type II collagen of the extracellular matrix. Its overexpression by chondrocytes correlates with cartilage degeneration and is recognized as an early marker of OA. Selective ablation of the *MMP-13* gene thereby suppresses the degradation of the extracellular matrix and reverses the degeneration process in OA. To knockout MMP-13 at the genome level, we designed a CRISPR/Cas9-based *MMP-13* targeted gene-editing system Cas9 sgMMP-13. The Cas9 sgMMP-13 includes a single guide RNA (sgRNA), which guides the Cas9 protein to the genomic locus of *MMP-13* (Figure 4A, Figure S4, and Table S1). The sgRNA sequence was inserted in the pSpCas9 plasmid to produce the Cas9 sgMMP-13 plasmid. The plasmid was mixed with Lipofectamine 2000 in an Opti-MEM medium at room temperature for 15 min and later incubated with exosomes for 12 h at 37 °C. The plasmid-loaded vesicles were then incubated with primary rat chondrocytes. The *MMP-13* level measured by qRT-PCR in chondrocytes indicated the *MMP-13* knockdown (Figure 4B). The hybrid CAP-Exo group exhibited the most effective suppression of the *MMP-13* level. These results suggest that the hybrid CAP-Exo is a more effective vehicle for the delivery of the Cas9 sgMMP-13 plasmid than others. To further prove that the surface proteins mediate the IA delivery of the hybrid CAP-Exo, we treated hybrid CAP-Exo with proteinase K, which non-selectively degrades all the proteins on the surface of exosomes. Proteinase K treatment significantly abolished the *MMP-13* inhibition (Figure 4C), indicating the surface proteins of the hybrid CAP-Exo play a critical role in chondrocyte delivery of the plasmid.

IL-1β is known to induce OA-like symptoms in chondrocytes, so we used IL-1β treatment to increase the expression level of *MMP-13* in chondrocytes to mimic the pathology of OA. Hybrid CAP-Exo loaded with the Cas9 sgMMP-13 plasmid significantly decreased the protein level of *MMP-13,* as shown by both immunoblotting and immunofluorescent studies using an anti-MMP-13 antibody (Figures 4D-E). Inhibition of *MMP-13* also suppresses collagen degradation. Using an anti-collagen II antibody, immunoblotting and immunofluorescent studies proved that knocking down *MMP-13* upregulated type II collagen (Figures 4D-E). These experiments show that the hybrid exosome-delivered Cas9 sgMMP-13 system knocks down *MMP-13* in chondrocytes and prevents the degradation of type II collagen, demonstrating the potential of hybrid exosomes in the treatment of OA. Sequencing analysis showed that a 182-nt sequence at the targeted *MMP-13* genomic loci was deleted (Figure S5). Lastly, we evaluated the cytotoxicity and hemolytic property of the carriers. The liposome showed some toxicity to chondrocytes (about 65% viability) and caused hemolysis to erythrocytes (3%). Neither Exo nor CAP-Exo showed noticeable cytotoxicity or hemolytic property, consistent with their cellular origin. Notably, the hybrid vesicles, hybrid Exo, and hybrid CAP-Exo also showed minimal cytotoxicity or hemolytic property (Figure S6). This data show that fusing exosomes with liposomes will mitigate the toxicity of the latter and give a biocompatible delivery vehicle.

### Intra-articular delivery of hybrid exosomes

**Figure 5:**
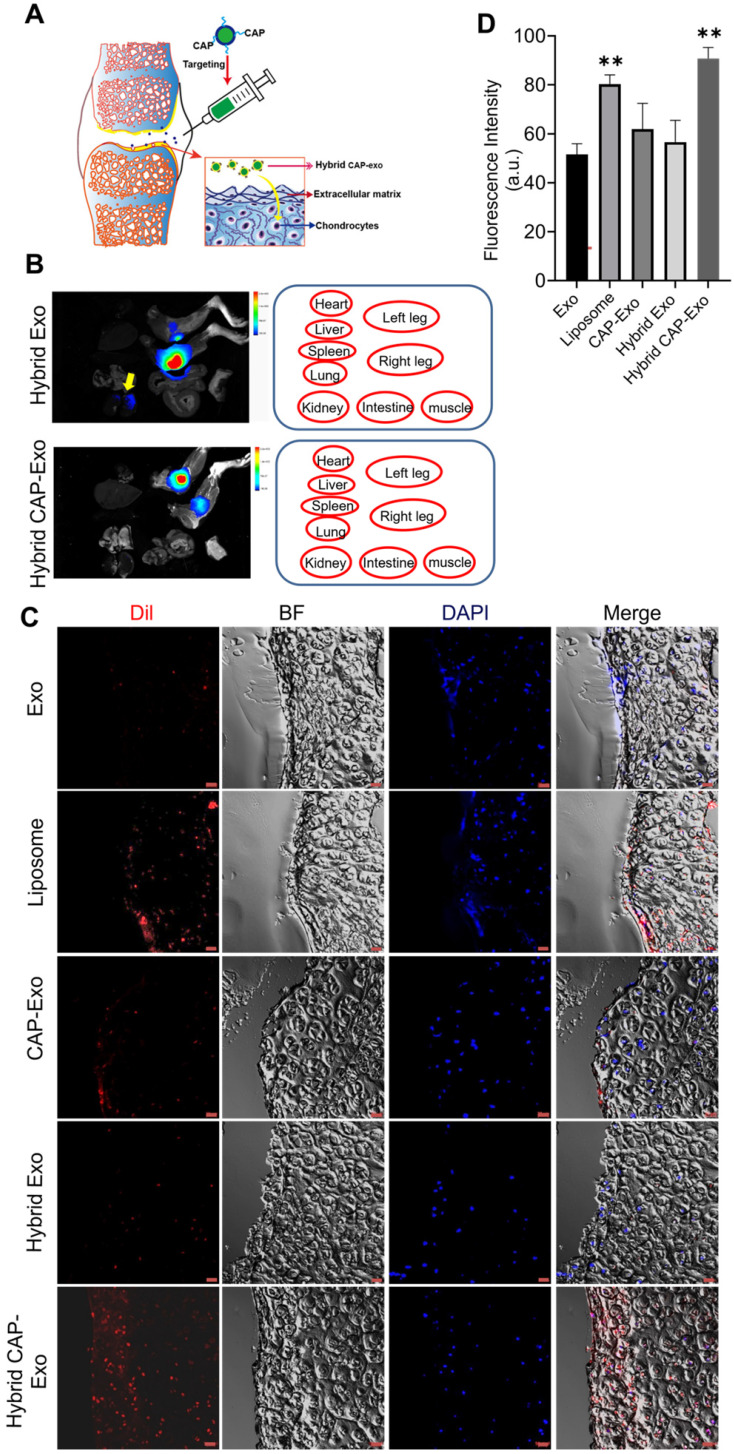
** Cartilage trafficking and chondrocyte internalization of IA-injected nanovesicles. (A)** Schematic illustration showing IA injection and nanovesicle uptake by chondrocytes. **(B)** Representative *ex vivo* fluorescence images of the major organs of rats after IA injection of DiI-labeled hybrid Exo or hybrid CAP-Exo. Nanovesicle were injected into the articular cavity of the knee joints. The positions of the organs in the fluorescent images are indicated in red circles. The yellow arrow points to the DiR signal in the kidney. **(C)** Representative confocal imaging shows the DiI signal of hybrid CAP-Exo and CAP-Exo penetrating deep into the rat cartilage. **(D)** Quantification of Dil-labeled nanovesicles in cartilage tissues in (C) (n = 3, means + SD). **p < 0.01 as compared to the Exo group. Scale bar, 20 µm.

After proving the efficacy of a hybrid CAP-Exo/Cas9 sgMMP-13 system in attenuating OA-related phenotype in a chondrocyte cell model, we next explored whether hybrid CAP-Exo can deliver the cargo through cartilage *in vivo* in a rat OA model. An expanded destabilization of medial meniscus (DMM) surgery was done to induce cartilage damage to mimic the condition of OA in 6-week SD rats. Hybrid exosomes were administered via IA injection into the knee joints of OA rats. Rats were sacrificed 1, 3, or 7 days after receiving the treatment. The knee joints were collected, fixed, decalcified, embedded in optimal cutting temperature (OCT) compound, and sliced (Figure 5A). The DiI signals were also found in the kidney for the hybrid Exo group, whereas in the hybrid CAP-Exo group, the signals were strictly confined to the knee joint (Figure 5B). Confocal images of the cartilage cryosections showed that hybrid Exo without the targeting CAP sequence were almost exclusively found on the surface of the cartilage after IA administration, whereas hybrid CAP-Exo were also found inside the matrix of the cartilage tissue where chondrocytes reside (Figures 5C-D). The signal of the hybrid CAP-Exo also lasted for at least 7 days in the cartilage (Figure S7). We also used a Cas9-GFP plasmid to validate the delivery efficiency. Higher GFP signals were observed in the chondrocytes using the hybrid CAP-Exo than in exosomes without CAP peptide (Figure S8). As the human cartilage (1 to 2 mm) is more than 10 times thicker than the rat cartilage, we next explored whether hybrid CAP-Exo can deeply penetrate human cartilage explants from OA patients. Labeled exosomes were incubated with human cartilage explants for 24 h before sectioning and visualization under confocal microscopy. Again, hybrid CAP-Exo showed the most significant penetration, accumulation, and retention in the human cartilage (Figure S9). All these results indicate that the CAP sequence displayed on the surface of exosomes endowed these nanovesicles with excellent capability to penetrate the cartilage matrix and retain within the cartilage for a prolonged period, thereby making hybrid CAP-Exo a promising chondrocyte-targeted delivery vehicle.

### CRISPR-mediated ablation of *MMP-13* alleviates OA

**Figure 6:**
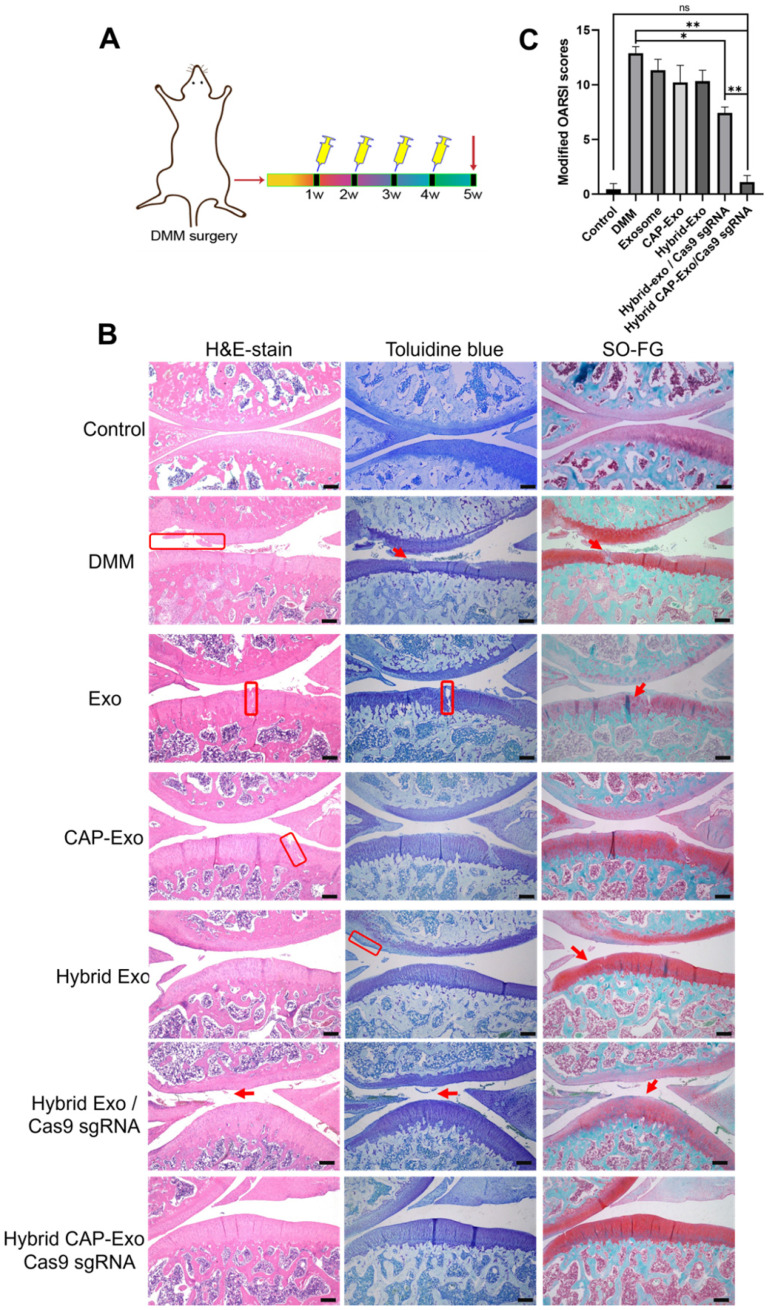
** IA injection of hybrid CAP-Exo/Cas9 sgMMP-13 reduced cartilage degeneration 4 weeks after surgical joint injury. (A)** Schematic illustration showing the experimental procedure. **(B)** Representative histological staining images of the cartilage tissues from different groups. **(C)** Modified OARSI scores of the cartilage tissues from different groups. Scale bar, 250 µm.

To translate our success in the OA cell model to animals, we next tested whether the hybrid CAP-Exo/Cas9 sgMMP-13 can alleviate OA symptoms in a rat model of OA. Similarly, DMM surgery was used to induce cartilage damage to mimic the condition of OA in SD rats. The treatment regime includes the injection of various therapeutic agents into the knee joint once per week for 4 weeks (Figure 6A). Hematoxylin and eosin (H&E) staining, toluidine blue staining, and Safranin O fast green (SO-FG) staining were used to evaluate the cartilage damage and proteoglycan loss. In the control, the blank group represents healthy rat cartilage with intact structure, clear layers, a smooth cartilage surface, and even staining. Significant cartilage damages were observed in the DMM group, including (1) coarse cartilage surface, indicating the erosion or degeneration of the cartilage layer (marked by red circles), (2) fractures of holes in the cartilage (marked by black arrows), and (3) insufficient staining or uneven staining, showing the loss of extracellular matrix. All the exosome-treatment groups, including Exo, CAP-Exo, hybrid Exo, and hybrid Exo/Cas9 sgRNA groups, showed a certain degree of improvement, but the damages remained to some extent. Only the hybrid CAP-Exo/Cas9 sgRNA treatment groups showed nearly identical imaging results to the control group, including a smooth cartilage surface, clear layers of the cartilage tissue, and an even staining of the dye (Figure 6B).

**Figure 7:**
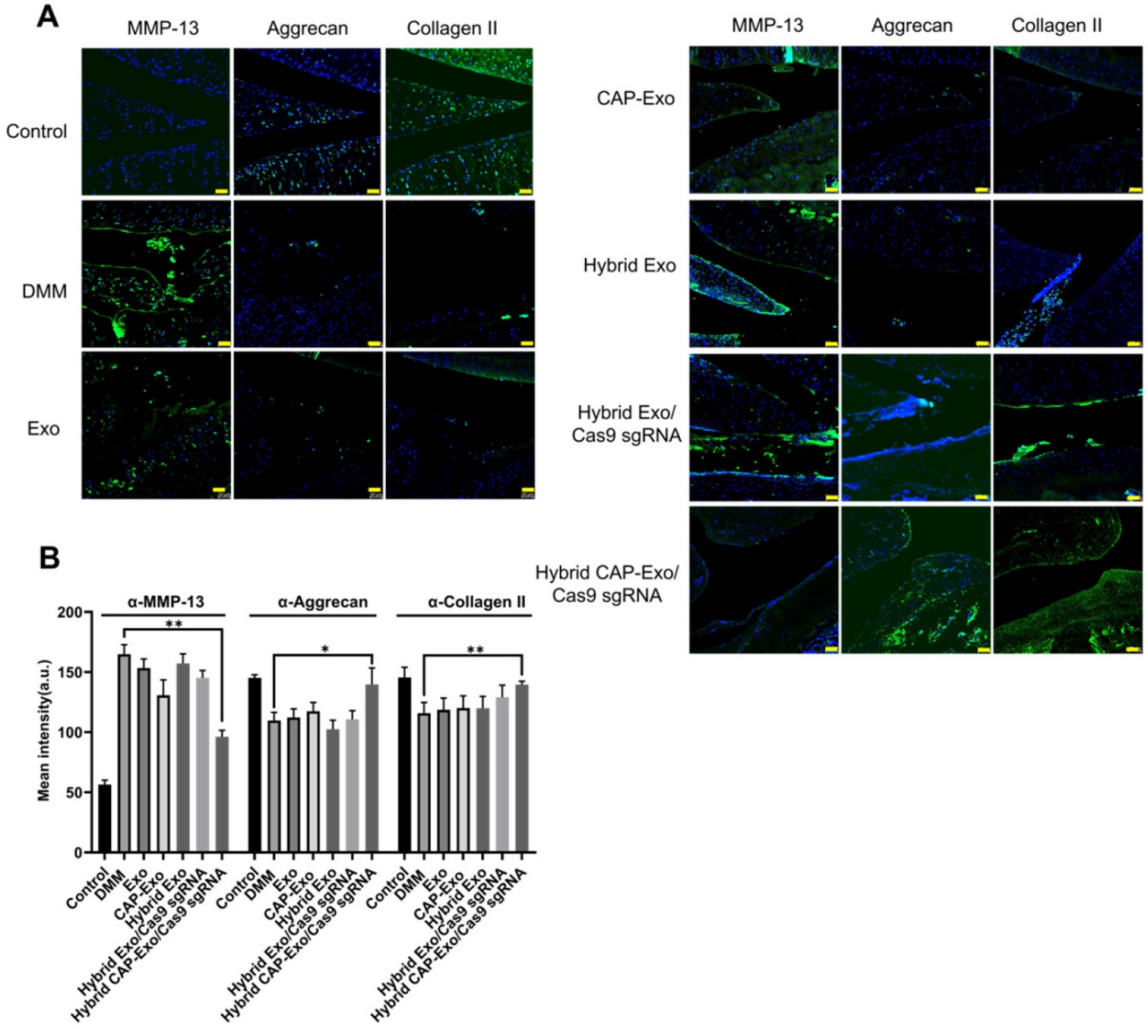
** Measurement of MMP-13, aggrecan, and collagen II levels in the knee joints. (A)** Representative immunofluorescence histochemistry staining images of the whole knee joint 4 weeks after DMM for the protein level of MMP-13, aggrecan, and collagen II in different treatment groups. Scale bar, 50 µm. **(B)** Quantification of the fluorescent signals in different groups.

We evaluated the results using the OARSI scoring system. The control group represents healthy cartilage giving the lowest score of 0.44. The DMM group received the highest score of 12.89, corresponding to a severe OA progression. All the treatment groups, including blank Exo, blank CAP-Exo, blank hybrid CAP-Exo, and hybrid Exo/Cas9 sgRNA show some improvement ranging from 7.44 to 11.33, suggesting that exosome alone does bring beneficial effect to the OA condition, likely through the anti-inflammatory effect of exosomes. However, the most remarkable result was seen in the hybrid CAP-Exo/Cas9 sgRNA group with the lowest score of 1.11, suggesting a nearly complete recovery (Figure 6C). Four weekly injections of chondrocyte-specific Cas9 sgMMP-13 inhibited the cartilage damage imposed by the DMM operation, evidenced by smooth cartilage surface, thick cartilage layer, uniform staining of the matrix, and no obvious cartilage degeneration as compared to the DMM group. Lastly, immunohistochemistry staining of MMP-13 and type II collagen in the whole knee joint confirmed that hybrid CAP-Exo/Cas9 sgMMP-13 treatment inhibited MMP-13 expression in cartilage in treated groups compared to the control group. Additionally, *MMP-13* knockdown also prevented collage II degradation and the decrease of aggrecan in cartilage (Figure 7).

## Conclusion

The delivery of the CRISPR/Cas9 tool into the target cells is a key step toward therapeutic genome editing. As delivery vehicles such as lentivirus and adeno-associated virus vectors raise concerns about immunogenicity and unwanted insertion into the genome, non-viral vesicles are likely a safer alternative 40. Here we demonstrate a strategy that combines both the feature of exosomes and liposomes to derive hybrid targeting exosomes for efficient delivery of the CRISPR/Cas9 plasmid to chondrocytes both *in vitro* and *in vivo*. Hybrid CAP-Exo retained the cell-specific targeting property of CAP-Exo, and fusion of exosome with Lipofectamine 2000 increased the stability and the capability of encapsulating plasmids. The affinity between exosomes and chondrocytes explains less organ diffusion, more prolonged cartilage localization, and deeper cartilage penetration. This is particularly important for OA treatment by IA administration, a convenient, minimally invasive method to directly target the therapeutics to the diseased sites of deteriorated cartilage. Although how deep hybrid CAP-Exo can penetrate human cartilage under the physiological condition still awaits further study, our hybrid CAP-Exo is likely the most suitable drug delivery vehicle for chondrocytes.

The study of OA has accumulated plentiful data on the genetics of this disease, and among all the genes associated with OA, a handful are druggable. MMP-13 is known as the major hydrolase that degrades type II collagen in the cartilage, which releases inflammatory factors and deteriorates the OA. Such an effect has not been found with other MMPs 41. This study proves that a CRISPR/Cas9 strategy of suppressing the expression of MMP-13 in chondrocytes shows remarkable therapeutic effect in OA models both *in vitro* and *in vivo*. This strategy is generally applicable to other genes associated with ECM degradation of cartilage. Screening these OA-related genes for the safest and most effective target with the help of the chondrocyte-targeted gene delivery system will pave a solid foundation for the potential treatment of OA that is minimally invasive, safe, and cell-free. Beyond the treatment of OA, this strategy may also be applied to cell-specific delivery of the genome editing tools for the treatment of other diseases. Although the long-term safety of genome editing in patients (including the immunogenicity of the plasmid DNA and Cas9 proteins and the potential off-target of the gene-editing) awaits further exploration, efficient and safe CRISPR genome editing is at its doorway to clinical uses and extracellular vesicles are certainly one of the promising vehicles 42, 43.

## Methods

### Plasmid construction

FLAG-Lamp2b plasmid was kindly gifted from Prof Matthew J. A. Wood's lab. To construct the CAP-Lamp2b vector, Xho1 and BspE1 cloning sites were first introduced to the targeting region in the N terminus of Lamp2b by polymerase incomplete primer extension (PIPE) cloning method.

pSpCas9(BB)-2A-Puro (PX459) containing Cas9 and sgRNA scaffolds was purchased from Addgene (plasmid #62988). Guide RNA (sgRNA) of rat *MMP-13* was designed using the online design tool available at http://crispr.genome-engineering. PX459 was digested with Bbs1 endonuclease, and a pair of oligos, including targeting sequences, were annealed and ligated to the BbsI-digested PX459 vector. The sgRNA sequences targeting MMP-13 are listed in Table S1.

### Exosome separation and purification

Dendritic cells were transfected with the CAP-Lamp2b plasmid bearing CAP-Lamp2b chimeric gene. Stable cells expressing this fusion protein were selected by G418. For exosome isolation, the cell culture medium was replaced with an exosome depleted FBS. Exosomes were isolated *via* centrifugation steps (300 × g for 10 min, 2 000 × *g* for 10 min, 10 000 × *g* for 30 min) and a filtration step through a 0.22 mm filter (Merck Millipore, Burlington, MA), then ultracentrifugation at 120 000 × *g* for 70 min. Exosomal pellets were re-suspended in PBS buffer.

### Exosome labeling

Exosomes (1 µg/µL) were labeled with DiI, or DiR, by incubation with the dyes (1 mM) at 37 ℃ for 30 min. Then unincorporated dye was removed by gel filtration using exosome Spin Columns (MW 3000, Invitrogen). For visualization of the exosomes by a confocal microscope, DiI-labeled exosomes were used. For *ex vivo* tracing of exosomes, DiR-labeled exosomes were used.

### Exosome-liposome hybridization and plasmid loading

Plasmid (5 μg) were mixed with Lipofectamine 2000 (1 mg/mL, 10 μl) in an Opti-MEM medium at room temperature for 15 min. Then exosomes were incubated with the mixture of liposomes and plasmids for 12 h at 37 °C.

### Exosome characterization

Nanoparticle tracking analysis (NTA). The size distribution of exosomes was measured using a nanoparticle tracking analysis instrument (NanoSight NS300, Malvern) after diluting samples 1000-fold with PBS.

Transmission electron microscopy (TEM). Exosomes were suspended in PBS and dropped on the copper grid. After the pellets were dried in the air, they were fixed with 3% (w/v) glutaraldehyde for 2 h and negatively stained with 2% uranyl acetate for 30 s. The samples were observed using a TEM operating at 300 kV (FEI TECNAI F30).

### Exosome uptake

To measure the internalization of exosomes into chondrocytes, equal amounts of DiI-labeled exosomes were added to cells in triplicate, and DMEM alone was added to control wells. Cells were incubated with DiI-labeled exosomes for 2 h. Images were acquired with Zeiss ZEN.

### Quantitative real-time PCR

Total RNA was extracted from cultured chondrocytes using TRIzol reagent (Invitrogen-Life Technologies, Carlsbad, CA). One microgram of extracted RNA was converted to cDNA using PrimeScript RT Reagent Kit (TaKaRa, Dalian, China), and Real-time quantitative PCR reactions were set up with Prime Script II RT-qPCR Kit (TaKaRa, Dalian, China) by the FastStart Universal SYBR Green Master (ROX) (Roche) using the CFX96 real-time system (Bio-Rad) employing primers in Table S1. Each sample was repeated 3 times and analyzed using GAPDH as the internal control.

### Chondrocyte cultures

Chondrocytes were isolated from the rat cartilage. Briefly, the cartilage sample was cut into pieces about 1 mm^3^ and treated with 0.5% collagenase II overnight. The mixture was filtered through the 100-μm cell strainer and cultured in Dulbecco's modified Eagle's medium/Ham's F-12 (DMEM/F-12) containing 10% FBS, 1% penicillin/streptomycin. After passage 3, chondrocytes were used for further *in vitro* experiments.

### Human OA cartilage explant harvest and culture

Human osteoarthritic cartilage samples were harvested from the surgical knee tissues of patients. Cartilage explants, 6 mm in diameter and 1-2 inches in thickness, were extracted from the cartilage samples using biopsy punches. Cartilage explants were cultured in DMEM-F12 (without phenol red), 10% (v/v) exosome-depleted FBS, and 1% (v/v) penicillin.

### Western blotting

Chondrocytes were cultured in DMEM/F-12 in the presence or absence of 5 ng/mL IL-1β. Cell lysates and conditioned medium were subjected to sodium dodecyl sulfate/polyacrylamide gel electrophoresis (SDS/PAGE) and immunoblotted with antibodies to MMP-13, Collage II, or GAPDH (Abcam, Cambridge, UK). To characterize specific markers of exosomes, the purified exosomes were resuspended in lysis buffer, and a cocktail of protease inhibitors was added (Roche). Western blotting was used to test positive markers (CD63, CD81, CD9, Alix) and negative markers (Calnexin). All images were acquired on an Odyssey imager.

### Proteinase K treatment

Exosomes were incubated with 100 μg/mL of Proteinase K (Roche Diagnostica GmbH, Germany) at 37 °C for 30 min. Then the exosomes were suspended in PBS solution and centrifuged at 120,000 × g for 1 h at 4 °C. Finally, the pellet was re-suspended in 200 μl PBS.

### OA animal experiments

Male SD rats were purchased from Zhuhai BesTest Bio-Tech Co., Ltd., Guangdong, China. Animal experiments were performed following ethical and statutory approval by Shenzhen TopBiotech Co., Ltd. OA induction was surgically induced in 6-week-old rats by expanded destabilization of the medial meniscus (DMM), which involves transecting the medial meniscotibial ligament and part of the meniscus under general anesthesia, as previously reported.

For *in vivo* fluorescence tracing of exosomes, after 1-week post-injury, rats were injected with 100 μg of DiR-labeled exosomes via intra-articular injection. The localization of the exosomes in different organs was detected by a Bruker Xtreme imaging system. Near-infrared fluorescence images of the whole animals and organs were obtained by a Bruker Xtreme imaging system.

For exosome tracing in cartilage explants, 100 ng DiI-labeled exosome was added to (6 mm×1inch ) cartilage explants. The explants were incubated for 48 hours at 37 °C and 5% CO_2_ under gentle agitation. After incubation, cartilage explants were washed three times with PBS, followed by embedding in OCT and cryosection into 5-µm sections using a vibrating microtome (Leica Biosystems). Quantitative analysis was performed on maximum intensity projections of z-stack images taken from 80-μm sections.

### Immunofluorescence analysis

Cells prepared for microscopy were cultured on confocal dishes. After 48-h post-transfection, cells were fixed in 4% paraformaldehyde in PBS (10 min). Cells were then rinsed twice in PBS and permeabilized with 0.2% Triton-X-100 in PBS. After washing with PBS, cells were blocked with 3% bovine serum albumin (BSA) in PBS (1 hour), washed three times with PBS, and incubated with 2-5 mg/mL primary antibody in PBS at 4 °C overnight. Following primary incubation, cells were washed three times (5 min) in PBS and incubated for 1 hour at room temperature with 1/250 dilution of highly cross-adsorbed secondary antibodies (goat anti-rabbit Alexa Fluor 488 or 594).

### Histopathology

Cryo-sectioning of cartilage tissues was done by following a standard procedure. Briefly, the tissue samples were thawed, mounted, and refrozen in OCT (optimum cutting temperature compound) embedding media, then cryo-sectioned into 6-μm thick sections using a Tissue-Tek II cryostat. The nuclei were stained with Hoechst 33342. Articular cartilage specimens from rats were fixed in 4% paraformaldehyde for at least 72 h and subsequently decalcified in EDTA buffer (20% EDTA, pH 7.4). Subsequently, tissues were embedded in paraffin. Serial sections (4 µm) were stained with hematoxylin & eosin (H&E staining). The sections were stained with hematoxylin-eosin-saffron, toluidine blue, and safranin O-fast green. Blinded assessment of histologic sections was done based on the Osteoarthritis Research Society International (OARSI) score system. For immunohistochemistry, the paraffin-embedded sections were processed and stained with rabbit anti-MMP-13 or anti-Collagen Ⅱ antibodies (Abcam, USA), followed by an Alexa Fluor 594® secondary antibody (Abcam, ab150080).

### Study approval

Collection of the cartilage explants from human OA knee joints was approved by the Ethics Committee of the First Affiliated Hospital of Shenzhen University, Shenzhen Second People's Hospital (20201109001-FS01). All the animal experiments were approved by the Ethics Committee of Shenzhen TopBiotech Co., Ltd (TOP-IACUC-2020-09-0010).

### Statistical analysis

Data are expressed as mean ± s.d. (mean ± standard deviation). Statistical significance between two groups was compared using Student's t-test, and one-way ANOVA was used to compare multiple groups by Tukey's post hoc test (GraphPad Prism software, version 8.02). *p*-values of Statistical analysis < 0.05 were considered statistically significant.

## Significance statement

The cartilage degeneration disease, osteoarthritis, has affected nearly 30 million people worldwide. A possible remedy is inhibiting the pathologically relevant essential proteins or genes in chondrocytes - the cells deeply embedded in the cartilaginous matrix. Delivering therapeutic agents into the chondrocytes remains a challenge due to difficulty crossing the dense cartilage tissue. Here we report a solution: we genetically engineered an exosomal surface protein Lamp2b to display a chondrocyte-affinity peptide on the surface of exosomes to allow the exosomes to target chondrocytes and retain in the joint after intra-articular (IA) injection. Furthermore, the fusion of exosomes with liposomes enables the hybrid exosome to encapsulate the CRISPR/Cas9 plasmid while maintaining the chondrocyte homing property. This strategy proves to be a valid cell-free, organelle-based therapeutic option for osteoarthritis. This approach is inexpensive, minimally invasive, highly effective, and holds promise to ease the pain of millions of osteoarthritic patients.

## Supplementary Material

Supplementary figures and table.

## References

[1] Patel MM, Patel BM (2017). Crossing the blood-brain barrier: recent advances in drug delivery to the brain. CNS Drugs.

[2] Liao W, Du Y, Zhang C, Pan F, Yao Y, Zhang T (2019). Exosomes: the next generation of endogenous nanomaterials for advanced drug delivery and therapy. Acta Biomater.

[3] Frangoul H, Bobruff Y, Cappellini MD, Corbacioglu S, Fernandez CM, De la Fuente J (2020). Safety and efficacy of CTX001 in patients with transfusion-dependent β-thalassemia and sickle cell disease: early results from the climb THAL-111 and climb SCD-121 studies of autologous CRISPR-CAS9-modified CD34+ hematopoietic stem and progenitor cells. Blood.

[4] Geurts MH, de Poel E, Amatngalim GD, Oka R, Meijers FM, Kruisselbrink E (2020). CRISPR-based adenine editors correct nonsense mutations in a cystic fibrosis organoid biobank. Cell Stem Cell.

[5] Dabrowska M, Juzwa W, Krzyzosiak WJ, Olejniczak M (2018). Precise excision of the CAG tract from the huntingtin gene by Cas9 nickases. Front Neurosci.

[6] Monteys AM, Ebanks SA, Keiser MS, Davidson BL (2017). CRISPR/Cas9 editing of the mutant huntingtin allele *in vitro* and *in vivo*. Mol Ther.

[7] Blanchard EL, Vanover D, Bawage SS, Tiwari PM, Rotolo L, Beyersdorf J (2021). Treatment of influenza and SARS-CoV-2 infections via mRNA-encoded Cas13a in rodents. Nat Biotechnol.

[8] Lin Y, Wu J, Gu W, Huang Y, Tong Z, Huang L (2018). Exosome-liposome hybrid nanoparticles deliver CRISPR/Cas9 system in MSCs. Adv Sci.

[9] McAndrews KM, Xiao F, Chronopoulos A, LeBleu VS, Kugeratski FG, Kalluri R (2021). Exosome-mediated delivery of CRISPR/Cas9 for targeting of oncogenic KrasG12D in pancreatic cancer. Life Sci Alliance.

[10] Duan L, Ouyang K, Xu X, Xu L, Wen C, Zhou X (2021). Nanoparticle delivery of CRISPR/Cas9 for genome editing. Front Genet.

[11] Duan L, Ouyang K, Wang J, Xu L, Xu X, Wen C (2021). Exosomes as targeted delivery platform of CRISPR/Cas9 for therapeutic genome editing. Chembiochem.

[12] Kim SM, Yang Y, Oh SJ, Hong Y, Seo M, Jang M (2017). Cancer-derived exosomes as a delivery platform of CRISPR/Cas9 confer cancer cell tropism-dependent targeting. J Control Release.

[13] Li Z, Zhou X, Wei M, Gao X, Zhao L, Shi R (2019). *In vitro* and *in vivo* RNA inhibition by CD9-HuR functionalized exosomes encapsulated with miRNA or CRISPR/dCas9. Nano Lett.

[14] Ye Y, Zhang X, Xie F, Xu B, Xie P, Yang T (2020). An engineered exosome for delivering sgRNA: Cas9 ribonucleoprotein complex and genome editing in recipient cells. Biomater Sci.

[15] Gee P, Lung MSY, Okuzaki Y, Sasakawa N, Iguchi T, Makita Y (2020). Extracellular nanovesicles for packaging of CRISPR-Cas9 protein and sgRNA to induce therapeutic exon skipping. Nat Commun.

[16] Lv Q, Cheng L, Lu Y, Zhang X, Wang Y, Deng J (2020). Thermosensitive Exosome-Liposome Hybrid Nanoparticle-Mediated Chemoimmunotherapy for Improved Treatment of Metastatic Peritoneal Cancer. Adv Sci.

[17] Rayamajhi S, Nguyen TDT, Marasini R, Aryal S (2019). Macrophage-derived exosome-mimetic hybrid vesicles for tumor targeted drug delivery. Acta Biomater.

[18] Xu X, Liang Y, Li X, Ouyang K, Wang M, Cao T (2021). Exosome-mediated delivery of kartogenin for chondrogenesis of synovial fluid-derived mesenchymal stem cells and cartilage regeneration. Biomaterials.

[19] Liang Y, Xu X, Li X, Xiong J, Li B, Duan L (2020). Chondrocyte-targeted microRNA delivery by engineered exosomes toward a cell-free osteoarthritis therapy. ACS Appl Mater Interfaces.

[20] Liang Y, Duan L, Lu J, Xia J (2021). Engineering exosomes for targeted drug delivery. Theranostics.

[21] Abramoff B, Caldera FE (2020). Osteoarthritis: pathology, diagnosis, and treatment options. Med Clin North Am.

[22] Hawker GA (2019). Osteoarthritis is a serious disease. Clin Exp Rheumatol.

[23] Jones G, Winzenberg T (2019). Osteoarthritis: a new short-term treatment option?. Lancet.

[24] Schmal H, Marintschev I, Salzmann GM (2016). Current status of anti-inflammatory therapy for posttraumatic osteoarthritis. Acta Orthop Belg.

[25] (2020). Drugs for osteoarthritis. Med Lett Drugs Ther.

[26] Richards MM, Maxwell JS, Weng L, Angelos MG, Golzarian J (2016). Intra-articular treatment of knee osteoarthritis: from anti-inflammatories to products of regenerative medicine. Phys Sportsmed.

[27] Chan HBY, Pua PY, How CH (2017). Physical therapy in the management of frozen shoulder. Singapore Med J.

[28] Zhao L, Huang J, Fan Y, Li J, You T, He S (2019). Exploration of CRISPR/Cas9-based gene editing as therapy for osteoarthritis. Ann Rheum Dis.

[29] Choi Y-R, Collins KH, Lee JW, Kang HJ, Guilak F (2019). Genome engineering for osteoarthritis: from designer cells to disease-modifying drugs. Tissue Eng Regen Med.

[30] Garnero P, Ayral X, Rousseau JC, Christgau S, Sandell LJ, Dougados M (2002). Uncoupling of type II collagen synthesis and degradation predicts progression of joint damage in patients with knee osteoarthritis. Arthritis Rheum.

[31] Piecha D, Weik J, Kheil H, Becher G, Timmermann A, Jaworski A (2010). Novel selective MMP-13 inhibitors reduce collagen degradation in bovine articular and human osteoarthritis cartilage explants. Inflamm Res.

[32] Hoshi H, Akagi R, Yamaguchi S, Muramatsu Y, Akatsu Y, Yamamoto Y (2017). Effect of inhibiting MMP13 and ADAMTS5 by intra-articular injection of small interfering RNA in a surgically induced osteoarthritis model of mice. Cell Tissue Res.

[33] Chen H, Qin Z, Zhao J, He Y, Ren E, Zhu Y (2019). Cartilage-targeting and dual MMP-13/pH responsive theranostic nanoprobes for osteoarthritis imaging and precision therapy. Biomaterials.

[34] Liang Y, Xu X, Xu L, Prasadam I, Duan L, Xiao Y (2021). Non-surgical osteoarthritis therapy, intra-articular drug delivery towards clinical applications. J Drug Target.

[35] Evans C (2016). Drug delivery to chondrocytes. Osteoarthritis Cartilage.

[36] Morgen M, Tung D, Boras B, Miller W, Malfait AM, Tortorella M (2013). Nanoparticles for improved local retention after intra-articular injection into the knee joint. Pharm Res.

[37] Rothenfluh DA, Bermudez H, O'Neil CP, Hubbell JA (2008). Biofunctional polymer nanoparticles for intra-articular targeting and retention in cartilage. Nat Mater.

[38] Alvarez-Erviti L, Seow Y, Yin H, Betts C, Lakhal S, Wood MJ (2011). Delivery of siRNA to the mouse brain by systemic injection of targeted exosomes. Nat Biotechnol.

[39] Salunkhe S, Dheeraj, Basak M, Chitkara D, Mittal A (2020). Surface functionalization of exosomes for target-specific delivery and *in vivo* imaging & tracking: Strategies and significance. J Control Release.

[40] Lino CA, Harper JC, Carney JP, Timlin JA (2018). Delivering CRISPR: a review of the challenges and approaches. Drug Deliv.

[41] Li H, Wang D, Yuan Y, Min J (2017). New insights on the MMP-13 regulatory network in the pathogenesis of early osteoarthritis. Arthritis Res Ther.

[42] Yao X, Lyu P, Yoo K, Yadav MK, Singh R, Atala A, Lu B (2021). Engineered extracellular vesicles as versatile ribonucleoprotein delivery vehicles for efficient and safe CRISPR genome editing. J Extracell Vesicles.

[43] Dooley K, McConnell RE, Xu K, Lewis ND, Haupt S, Youniss MR (2021). A versatile platform for generating engineered extracellular vesicles with defined therapeutic properties. Mol Ther.

