# Supplementary material for: Chondrocyte-specific genomic editing enabled by hybrid exosomes for osteoarthritis treatment
Source: Theranostics. 2022 Jun 21;12(11):4866–78. doi: 10.7150/thno.69368 (PMC9274754; doi:10.7150/thno.69368)
Supplement: Supplementary file 1 — Supplementary figures and table. [file thnov12p4866s1.pdf]

Supporting Information

Chondrocyte-specific genomic editing enabled by hybrid exosomes for osteoarthritis treatment

Yujie Liang[a,b, †], Xiao Xu[a, †], Limei Xu[a, †], Kan Ouyang[a], Huawei Zhang[c], Chunyi Wen[d, e], Li Duan[a,*], Jiang Xia[b,*]

[a] Department of Orthopedics, the First Affiliated Hospital of Shenzhen University, Shenzhen Second People's Hospital, Shenzhen 518035, China.

[b] Department of Chemistry, the Chinese University of Hong Kong, Shatin, Hong Kong SAR, China.

[c] Department of Biomedical Engineering, South University of Science and Technology of China, Shenzhen, 518055, China.

[d] Department of Biomedical Engineering, [e] Research Institute of Smart Ageing
The Hong Kong Polytechnic University, Hong Kong SAR, China.

[†]Y. L., X. X., and M. Li contributed equally to this work.

*To whom correspondence may be addressed. Email: jiangxia@cuhk.edu.hk, whjy2004@gmail.com.

Contents

Items	Pages
**Table S1.** Sequences of sgRNA and primers for qRT-PCR analysis	3
**Figure S1**. Zeta potential of different nanovesicles.	4
**Figure S2.** Evidence of the fusion of exosome and liposome fusion based on FRET data.	5
**Figure S3.** Representative flow cytometry histograms showing the uptake of different nanovesicles.	6
**Figure S4**. The design, encapsulation, and selection of Cas9 sgMMP-13.	7
**Figure S5**. Evidence of genome editing by sequencing analysis showing the deletion of a 182-nt sequence at the targeted MMP-13 genomic loci by Cas9/SgMMP13 #1.	8
**Figure S6**. Biocompatibility of the vesicles evaluated by cytotoxicity and hemolytic properties.	9
**Figure S7.** Significantly longer retention of the targeting hybrid exosomes in the cartilage.	10
**Figure S8**. Comparison of GFP signal as the result of vesicle-delivered Cas9-GFP plasmid in the rat cartilage *in vivo.*	11
**Figure S9**. Exosomes penetrate cartilage explants from osteoarthritic patients.	12

Table S1. Sequences of sgRNA and primers for qRT-PCR analysis.

sgRNA1-MMP-13-F	CACCGGGACCATTTGAGTGTTCGA
sgRNA1-MMP-13-R	AAACTCGAACACTCAAATGGTCCC
sgRNA2-MMP-13-F	CACCTCCTGGACCAAACCTTGGCG
sgRNA2-MMP-13-R	AAACCGCCAAGGTTTGGTCCAGGA
qRT-MMP-13-f	CTGACCTGGGATTTCCAAAA
qRT-MMP-13-r	ACACGTGGTTCCCTGAGAAG
qRT-GAPDH-f	TGACTTCAACAGCAACTC
qRT-GAPDH-r	TGTAGCCATATTCATTGTCA
gMMP-13-f	TGGCTTAGATGTGACTGGC
gMMP-13-r	CATCATAAATCTAGCATA

Note: sgRNAs targeting exon of *MMP-13* were designed at www.crispr.mit.edu. Synthesized sgRNA 1 and sgRNA 2 were individually subcloned into pSpCas9(BB)-2A-Puro (PX459) V2.0 vector. pSpCas9(BB)-2A-Puro (PX459) V2.0 was obtained from Feng Zhang (Addgene plasmid # 62988).

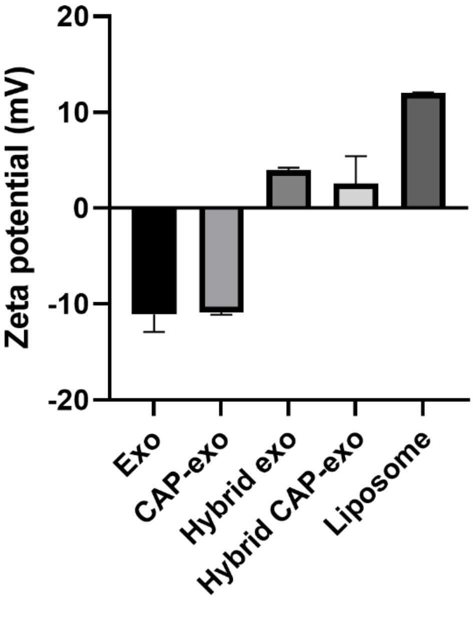

Figure S1. Zeta potential of different nanovesicles.

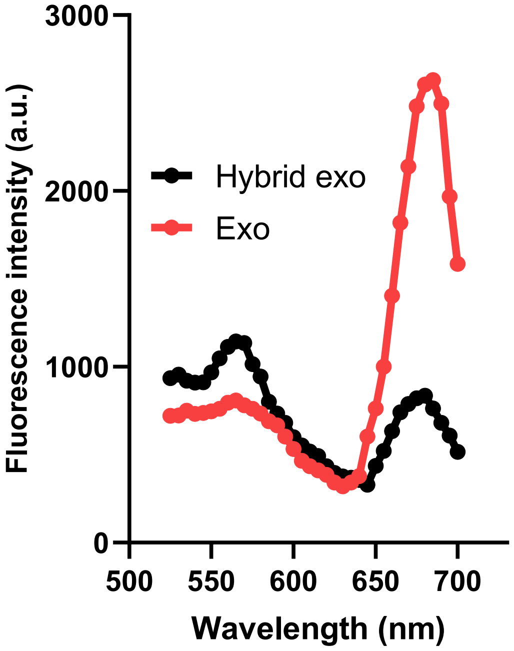

Figure S2. Evidence of the fusion of exosome and liposome fusion based on FRET data. Briefly, exosomes (1 mg/mL) were double-labeled with lipophilic fluorescent dyes Vybrant DiI (Molecular Probes, V-22885) and DiD (Molecular Probes, V-22887) and then filtered through a 100 kDa Ultra Centrifugal Filter Unit (Amicon®) to remove free dyes. Under a fluorescent spectrometer (BioTek Instruments, USA), a strong emission peak was observed at around 685 nm (λem = 488 nm), showing that both DiI and DiD dyes were enriched in the membrane (red dotted line). Mixing liposomes with labeled exosomes increased the emission at 565 nm and decreased the emission at 685 nm (black dotted line). The decreased FRET effect was the fusion of exosome and liposome which extended the surface area of the vesicles and enlarged the distance between DiI and DiD.

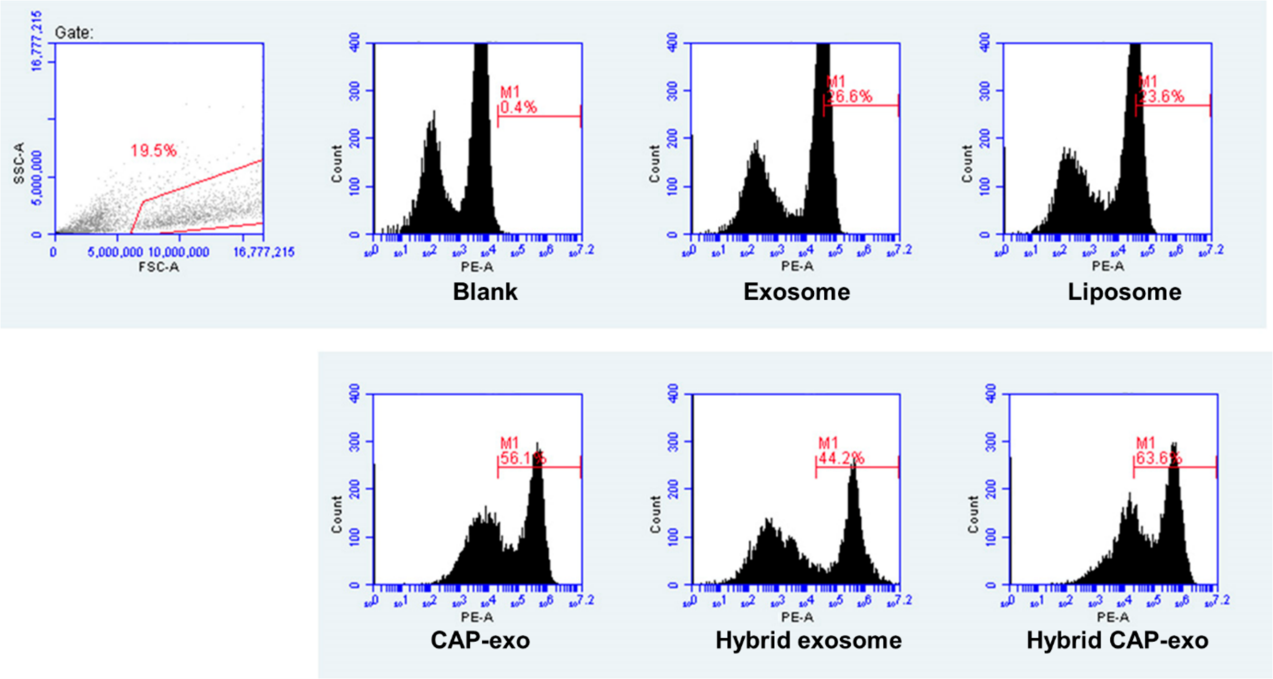

Figure S3. Representative flow cytometry histograms showing the uptake of different nanovesicles. Nanovesicles with surface-displayed CAP sequence showed significantly higher uptake in rat chondrocytes (>50%) than nanovesicles without CAP. Briefly, DiI-labeled exosomes or hybrid exosomes (10 μg/ml) were labeled with DiI and incubated with rat chondrocytes, washed, and harvested for subsequent flow cytometry analysis.

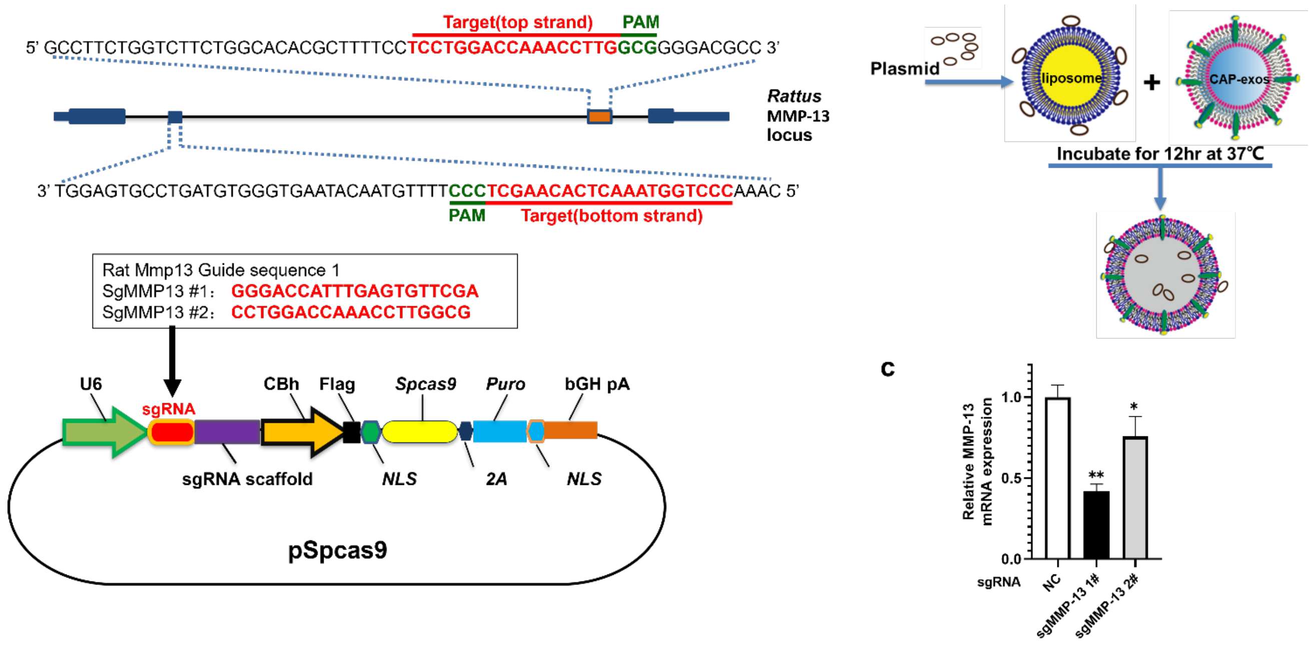

Figure S4. The design, encapsulation, and selection of Cas9 sgMMP-13. (a) Details of the Cas9 sgMMP-13 and its packaging in pSpcas9 plasmid. Briefly, synthesized SgMMP13 #1, GGGACCATTTGAGTGTTCGA and SgMMP13 #2, CCTGGACCAAACCTTGGCG were individually subcloned into pSpCas9(BB)-2A-Puro (PX459) V2.0 vector (Addgene plasmid # 62988). (b) Schematic illustration showing how Cas9 sgMMP-13 was encapsulated in hybrid exosome. Briefly, Lipofectamine 2000 and the pSpcas9 plasmid were mixed and incubated at room temperature for 15 min. Then, the complex was added to exosomes and incubated at 37 °C for 12 h. (c) Two sgRNAs targeting different loci of the *MMP-13* gene were evaluated based on the expression level of *MMP-13* in chondrocytes. One of the Cas9 sgMMP-13 constructs, using sgMMP-13 #1 gives more significant reduction of the *MMP-13* level and thus was selected for the following experiments.

a

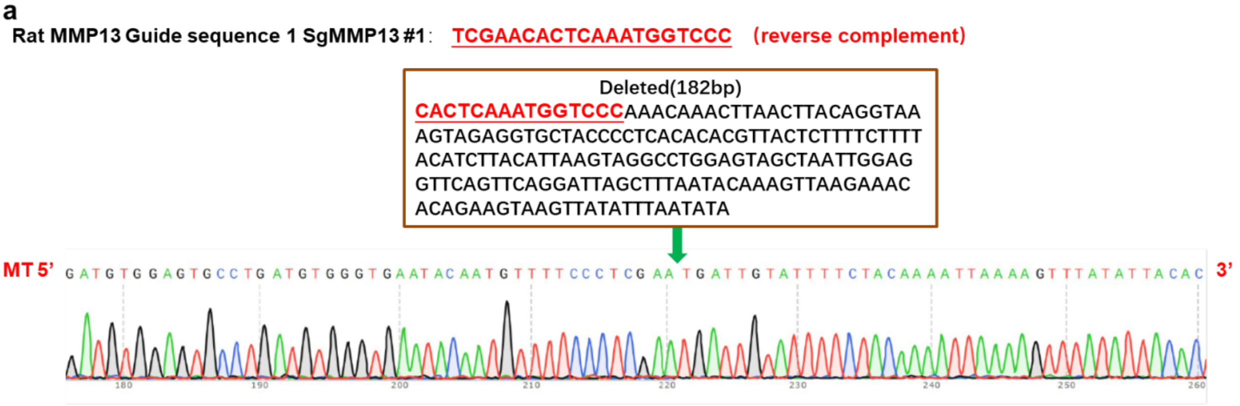

b

AAACCAAGATGTGGAGTGCCTGATGTGGGTGAATACAATGTTTT*CCCTCGAACACTCAAATGGTCCC*AAACAAACT
TAACTTACAGGTAAAGTAGAGGTGCTACCCCTCACACACGTTACTCTTTTCTTTTACATCTTACATTAAGTAGGCCTG
GAGTAGCTAATTGGAGGTTCAGTTCAGGATTAGCTTTAATACAAAGTTAAGAAACACAGAAGTAAGTTATATTTAAT
ATATGATTGTATTTTCTACAAAATTAAAAGTTTATA

Figure S5. Evidence of genome editing by sequencing analysis showing the deletion of a 182-nt sequence at the targeted MMP-13 genomic loci by Cas9/SgMMP13 #1. (a) Sequencing result showing the deletion of a 182-nt sequence. (b) Overall sequence showing the position of the 182-nt fragment. The red sequence is the SgMMP13#1. The blue sequence represents the 182-nt fragment that is missing. Briefly, rat chondrocytes were stably transfected with CRISPR/Cas9-SgMMP13 #1, and then genomic DNA was extracted. The genomic sequence containing the target sites of MMP-13 was amplified from the genomic DNA by using specific primers (gMMP-13-f and gMMP-13-r), and the PCR fragments were selected for subsequent sequenced to identify the mutation site. Deletion of a 182-nt sequence was observed.

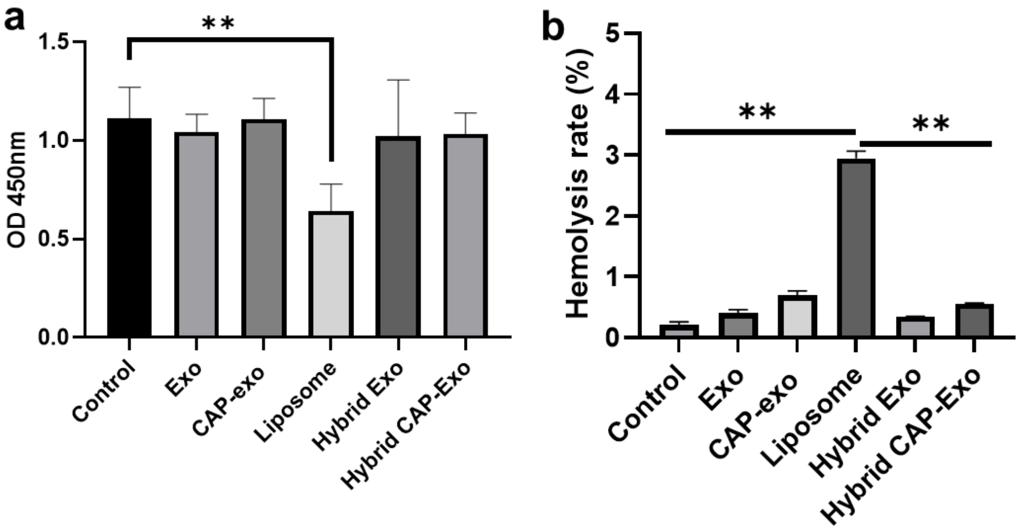

Figure S6. Biocompatibility of the nanovesicles evaluated by cytotoxicity and hemolytic properties. (a) Measurement of the cytotoxicity by CCK8 assay. Briefly, rat chondrocytes were seeded in 96-well plates (2×10^4 cells/mL, 100 μL) and incubated for 24 h. DMEM with exosome-free FBS was used as the control. The cells were then incubated with vesicles for 24 h and cell viability was determined using the CCK8 assay. (b) Nanoparticle affect the hemolysis rate of human erythrocytes. Data were expressed as mean ± SD of three different experiments. *p < 0.05, **p < 0.01.

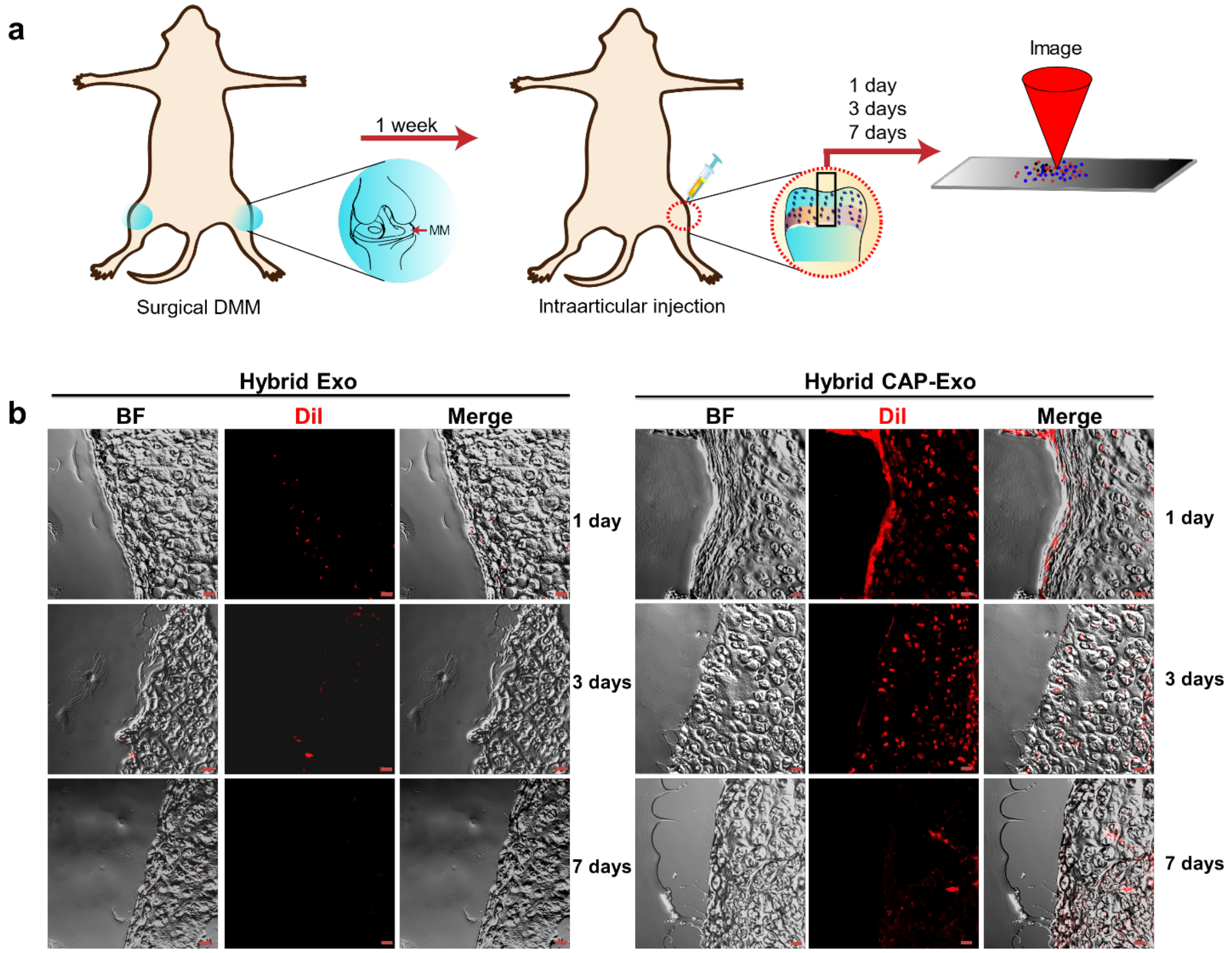

Figure S7. Significantly longer retention of the targeting hybrid exosomes in the cartilage. Rats received DMM surgery received intraarticular injection of DiI-labeled hybrid exosome without targeting CAP or hybrid CAP-Exo respectively. The fluorescence signals inside the cartilage capsule were observed under the confocal microscope. The hybrid CAP-Exo showed longer retention. BF: bright field. Scale bar, 20 μm.

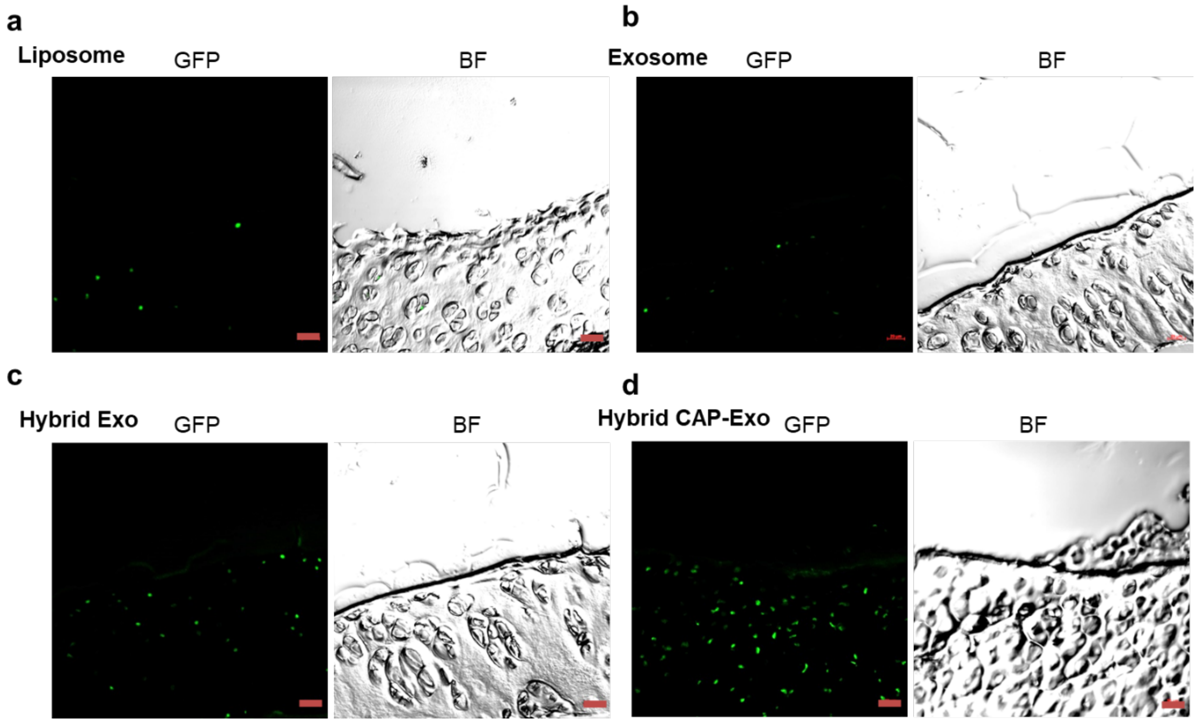

Figure S8. Comparison of GFP signals as the result of vesicle-delivered Cas9-GFP plasmid in the rat cartilage *in vivo*, (a) liposome, (b) exosome, (c) hybrid exo, and (d) hybrid CAP-exo. The hybrid CAP-exo group shows the highest GFP signal, suggesting the highest delivery efficiency assuming each group has the same expression level of Cas9-GFP. Scale bar, 20 µm. Briefly, rats that underwent DMM surgery were intraarticularly injected with hybrid exosome, exosome, or liposome that has been preincubated with the Cas9-GFP plasmid. The expression of GFP fluorescence signals inside the cartilage capsule was detected by a confocal microscope.

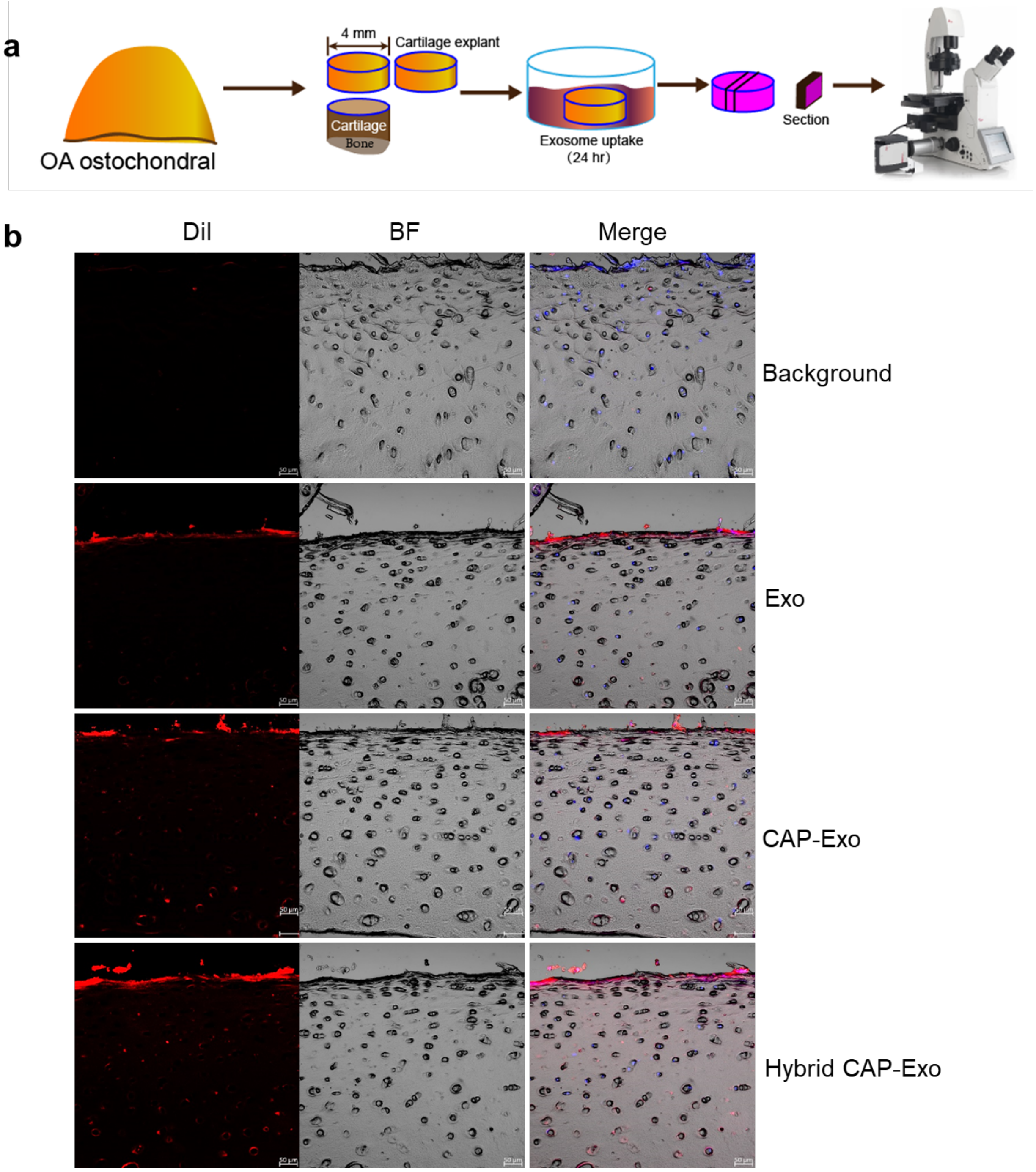

Figure S9. Exosomes penetrate cartilage explants from osteoarthritic patients. (a) Schematic illustration of the cartilage explant assay. Briefly, cartilage explants were harvested from human OA patients. After incubated with DiI labeled exosome or hybrid exosome for 24 h, cartilage explants were washed three times with PBS, followed by embedding in OCT, and explant slices were sectioned in the x-y plane, the confocal microscopy was used to visualize the transport of exosome into the explant disks in the x-direction at selected times. (b) Representative fluorescent images of DiI-labeled exosomes at different time points following the exosome incubation. Scale bar, 20 μm.
